# Evaluation of Physicians’ Knowledge and Attitudes Towards Biosimilars in Russia and Issues Associated with Their Prescribing

**DOI:** 10.3390/biom9020057

**Published:** 2019-02-11

**Authors:** Dmitry Karateev, Natalia Belokoneva

**Affiliations:** 1 Rheumatology Department, Moscow Regional Research and Clinical Institute, Shchepkina, 61/2, Moscow 129110, Russia; dekar@inbox.ru; 2 Pfizer Ltd., Maidenhead, Berkshire SL6 6RJ, UK

**Keywords:** awareness, biosimilars, education, physicians, perceptions, Russia

## Abstract

Physician awareness and perceptions towards biosimilars are important factors in their adoption to clinical practice. Our objectives were to assess levels of knowledge and attitudes towards biosimilars and key policies on their use among Russian physicians, define the level of interest in new information on biosimilars, and determine what evidence drives treatment decisions in Russia. Physicians with awareness of biologics across different specialties and regions of Russia completed an online survey. A Likert and other rating scales were used to collect opinions, which were summarized descriptively. Responses of subgroups of respondents were compared using *t*-tests. Among 206 respondents (*n* = 51 rheumatologists; *n* = 53 gastroenterologists; *n* = 50 hematologists; *n* = 52 oncologists), 66% had positive impressions regarding the introduction of biosimilars in Russia. Overall, 80% lacked understanding of the differences between biosimilars and generics. In all, 67% supported prescribing biologics by distinguishable names and were negative about tender policies limiting choice of therapies for patients. The majority believed in mandatory publication of clinical trial results on biosimilars (94%), agreed biosimilars should be subject to rigorous post-marketing surveillance (98%), and expressed willingness to learn more about biosimilars (94%). Biosimilar education among Russian physicians is required, which may help shape balanced and evidence-based policies for biosimilars in Russia.

## 1. Introduction

Biologic therapies have revolutionized treatment for many life-threatening and debilitating diseases; however, patient access to these medicines can be restricted [1]. The recent expiry of patent portfolios for the first innovator biopharmaceuticals (reference products) has led to the development and authorization of similar biological medicinal products, known as biosimilars [2,3]. Biosimilars are biologic products that are highly similar to a licensed reference biologic with respect to their quality characteristics, pharmacology, efficacy and safety, such that there are no meaningful differences between the biosimilar and reference product when used in clinical practice [4,5,6]. Since biosimilars are similar, but not identical, to their reference products, they cannot be considered generic versions of biologic drugs. Consequently, the regulatory approval process for generic medicines is not applicable to biosimilars [7]. 

The European Medicines Agency and World Health Organization (WHO) [4,5], as well as a number of countries [6,8], have issued guidance on biosimilar regulatory pathways. Approval of biosimilars requires comprehensive assessment of all stages of the research and development process, including evaluation of analytical, preclinical and clinical data, to establish biosimilarity to their reference products. The goal of biosimilar comparability studies is not to re-establish efficacy and safety for the proposed biosimilar, but to demonstrate similarity to the reference product [4,5,6]. 

In Russia, the regulatory and policy landscape for biosimilars is different to countries/regions where biosimilar guidelines are firmly established. Russia does not yet have regulatory guidelines for biosimilars. However, ≈50 biosimilars, including four monoclonal antibodies, have been approved in Russia despite a lack of guidelines for biologic products and by using an approach akin to those for small-molecule generic drugs [9,10,11,12,13]. It has also been established that a full clinical development program must be completed before the registration of biologic drugs [10,11,12,13,14,15]. Additionally, in recent years, definitions for biologic products have been introduced [16]. For example, the terms “bioanalog” and “reproduced drug” are used instead of “biosimilar” because there is no suitable translation into Russian. The regulatory definition is: “Bioanalogic (bio-like) medicinal product (bioanalog) is a biological medicinal product similar in quality, efficacy, and safety parameters with a reference biological drug in the same dosage form and having an identical mode of administration” [16]. Additionally, regulators in Russia have established that a biosimilar may be recognized as interchangeable at the stage of their registration based on the demonstration of biosimilarity, without any specific requirements to examine multiple switches between the reference product and the biosimilar candidate within the clinical program (as the recent US Food and Drug Administration (FDA) draft guidelines requires), and without any requirements for post-marketing data [16]. An “interchangeable medicinal product” is defined as: “a medicinal product with a proven therapeutic equivalence or bioequivalence with respect to a referent medicinal product having an equivalent qualitative composition and a quantitative composition of the active substances, an adjuvant composition, a dosage form and a mode of administration” [16].

In Russia, physicians are required to prescribe biologic medicines using the international non-proprietary names (INNs) [17]. Use of INNs means that two or more medicines (the originator biologic medicine and all approved biosimilars) can share the same INN. In the context of biologic medicines, this may result in the unintended switching of original biologics with a biosimilar, or a biosimilar with another biosimilar. This practice carries inherent risks and may confound pharmacovigilance [11,18,19,20,21]. The WHO proposed that a biosimilar’s name and labeling should be distinguishable from the reference product and that biosimilars should be subject to rigorous post-marketing surveillance [22].

State procurement of biologic medicines in Russia is via winner-takes-all tenders [17]. Local manufacturers also have 15% price preferences in tenders versus international companies; therefore, physicians often have only one locally produced biosimilar, and not a reference product, available for prescription [23]. Additionally, a Russian government decree requires that clients must decline applications for the supply of medicines of foreign origin (excluding the Eurasian Economic Union (EAEU) member-states) if there are two other applications available to supply medicines produced in the EAEU member-states [24]. Other issues specific to Russia include the absence of transparency in results of locally conducted clinical trials [25]. Some biosimilars have been approved in Russia using a mainly generic approach [10,11,13]. However, recently, some biosimilars have been evaluated in comparative clinical trials with originator (reference) products [26,27,28]. It must be noted that at the time this survey was conducted, data were not available for some biosimilars that have now been approved in Russia (Table S1). Additionally, there is a legal requirement in Russia, for medical organizations to be licensed for the treatment of patients with biologics (in so-called “specialized health care” and “high-technology specialized health care” settings).

To date, there are no published data about physicians’ knowledge and attitudes towards biosimilars in Russia or the issues associated with prescribing biosimilars. Understanding physicians’ attitudes and perceptions can help to develop future educational programs and highlight important issues for payers, policymakers and other stakeholders. Therefore, the objectives of this study were to survey the knowledge and attitudes of Russian physicians towards biosimilars and related policies, define the level of interest in information on biosimilars, and determine what evidence drives treatment decisions in Russia.

## 2. Materials and Methods

### 2.1. Questionnaire and Recruitment 

A comprehensive questionnaire for self-completion was developed in English by Natalia Belokoneva (Survey Questionnaire 1) [29,30,31]. The questionnaire was translated (Survey Questionnaire 2) and the survey conducted in native (Russian) language from 15 June to 22 July 2016. The questionnaire contained 15 questions (unrelated to any particular product) based on publicly available surveys on biosimilars conducted in other countries [29,30,31], with the addition of questions to address country-specific issues. 

A database for recruitment was developed and included clinicians from a range of specialties (rheumatology, gastroenterology, hematology, and oncology) who used biologic therapies from hospitals and centers across Russia. Physicians were recruited via email and those who agreed to participate were provided with an online questionnaire that included both closed-ended and open-ended questions, to collect answers from a number of perspectives. Physicians had the opportunity to enter comments after most questions and at the end of the survey. The questionnaire did not distinguish between academic and community-based physicians, nor did it capture the years of practice for each physician. To encourage participation in the survey, the participants’ responses were anonymous and no personal information was collected; therefore, ethical research committee approval was not required [32]. 

The number of Russian physicians who are eligible to prescribe for and manage patients receiving biologic therapy is low and they are mostly located in medical centers and hospitals, to which patients are referred when requiring treatment with biologic medicines. Therefore, before providing the online survey, a telephone interview (lasting approximately 15 min) was conducted to ensure participants had experience with biologic medicines and were familiar with biosimilars (self-assessed) (see Survey Questionnaire 1, questions S1–S4). Due to the complex process of licensing medical organizations, only a limited number are authorized to initiate treatment with biological medications. For this reason, in medical centers specializing in biologic treatment, physicians were invited to participate in the survey by a “snowball-sampling” technique (i.e., based on the recommendations of participating colleagues at the time of the telephone interview) [33]. All screened-in physicians were invited to participate by email until 206 physicians were surveyed. The questionnaire was issued to all participants simultaneously and they were given one month to complete it; follow-up emails were sent to request return of the completed survey, if required.

Recruitment and data collection were performed with support of the Ipsos marketing agency (Moscow, Russia) and sponsored by Pfizer LLC (Moscow, Russia). Pfizer Inc. identified the specialized centers authorized for the treatment of patients with biologics, from which Ipsos developed the database of physicians for recruitment. Respondents who completed the questionnaire received remuneration from Ipsos for their participation. The online questionnaire was developed using IBM SPSS Data Collection 6 software package (IBM Corporation, Armonk, NY, USA). Only fully completed questionnaires were included in the analyses.

### 2.2. Analyses

A sample size of 50 clinicians per specialty (rheumatology, gastroenterology, hematology, and oncology) was considered sufficient for the study objectives and was in alignment in size with similar studies conducted in other countries [29,30]. Furthermore, to the estimated sample size was consistent with the central limit theorem, such that, regardless of the distribution of the sampling population, if the sample size is sufficiently large (*n* ≥ 30), then the population of all possible sample means is approximately normally distributed. The larger the sample size, the more nearly normally distributed is the population of all possible sample means [34]. Since the general population of clinicians prescribing biologics and revealing familiarity with biosimilars was homogeneous and did not have strong asymmetry, a sample size of *n* = 50 per specialty was deemed to meet the study objectives. A Likert scale and other rating scales were used to collect opinions and convert them into a numerical format, which were then summarized descriptively. Subgroups of respondents were compared using *t*-tests. A *p*-value < 0.05 was considered statistically significant. To correct over- and under-representation of specialties after pre-screening, a weighting-adjustment technique was applied. Weighted values were used to compute all the descriptive statistics [35]. The software package IBM SPSS Statistics 13 (IBM Corporation, Armonk, NY, USA) was used to analyze numerical and categorical data.

## 3. Results 

### 3.1. Participating Physicians

Among 210 physicians who were screened by telephone, 43% had prescribed biologic therapies in the past 12 months and were familiar with biosimilars. One-fifth neither prescribed biologics nor were familiar with biosimilars (Figure S1). Of the specialties, hematologists (79%) and gastroenterologists (23%) prescribed the greatest and least number of biologic therapies, respectively. Overall, 57% of respondents had experience with biologic treatments and 66% were “somewhat familiar”, “familiar” or “very familiar” with biosimilars. The level of familiarity with biosimilars was equally distributed across specialties.

Of the 210 physicians who were screened by telephone, 81 (39%) had experience with biologic medicines and were familiar with biosimilars (self-assessed) and 70 completed the questionnaire. An additional 136 physicians were recruited by the snowball-sampling technique, all of whom had experience with biologic medicines and were familiar with biosimilars (self-assessed), and all completed the survey. Therefore, a total of 206 respondents (70 + 136 physicians) were included in the analysis (Figure S2). Of these 206 respondents, 51 were rheumatologists, 53 were gastroenterologists, 50 were hematologists, and 52 were oncologists. The study covered nine Russian cities; most respondents were from Moscow (36%), followed by St. Petersburg (16%). 

### 3.2. Knowledge of Biosimilars and Familiarity with Their Regulation in Russia

**Figure 1:**
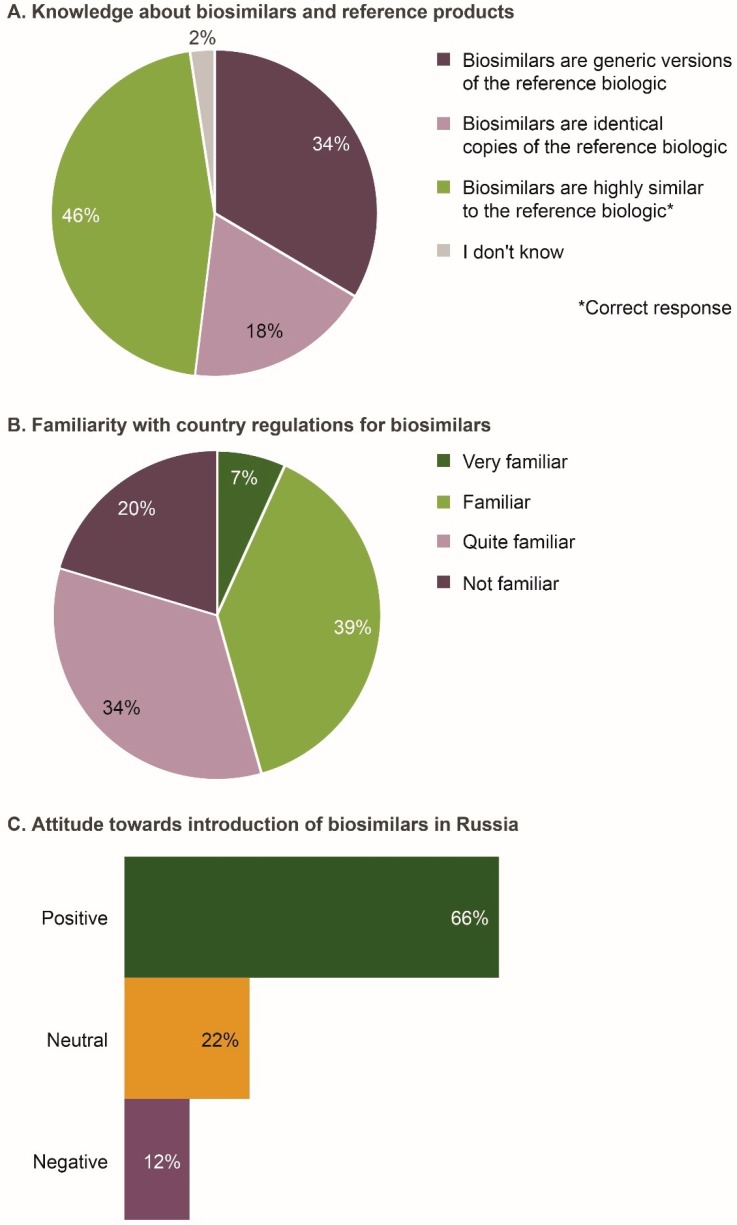
Level of understanding of biosimilars by the Russian physicians surveyed (*n* = 206). (**A**) Knowledge about biosimilars and reference products; (**B**) Familiarity with country regulations for biosimilars; (**C**) Attitude towards introduction of biosimilars in Russia. Data were extracted from questions 4, 5, and 7e of the questionnaire (see Survey Questionnaire 1).

Of the 206 respondents, 46% correctly defined biosimilars to be highly similar versions of their reference products (Figure 1A). However, in response to a separate question later in the questionnaire, 37% agreed that biosimilars were the same as generic drugs. Based on the two questions, only 20% of individuals twice confirmed that biosimilars were different from generic drugs and that they were not identical copies of reference products. 

Overall, 46% of respondents indicated that they were familiar/very familiar with the approval pathway and Russian regulations for biosimilars (Figure 1B). No significant differences were observed across specialties and geographic regions (Table S2). 

The majority of respondents (66%) were positive regarding the introduction of biosimilars in Russia (Figure 1C). Compared with other specialties, fewer gastroenterologists (2%; *p* = 0.001) were negative about the introduction of biosimilars. Moreover, 91% of respondents agreed/strongly agreed that they would be comfortable treating patients with a biosimilar if equivalent safety and efficacy had been demonstrated in a well-designed comparative trial. Respondents cited affordability, increased patient access to biologic medicines, increased competition in development and commercialization of biologics, and increased treatment options, as potential benefits of biosimilars. Some physicians stated that they had had a positive clinical experience with biosimilars. Reasons for a neutral or negative attitude towards biosimilars included not understanding the rationale for extrapolation, lack of experience, and believing locally produced biosimilars to be of lower clinical efficacy, safety and quality than internationally produced biosimilars. 

### 3.3. Attitudes Towards Key Policy Issues Associated with Prescribing Biosimilars 

**Figure 2:**
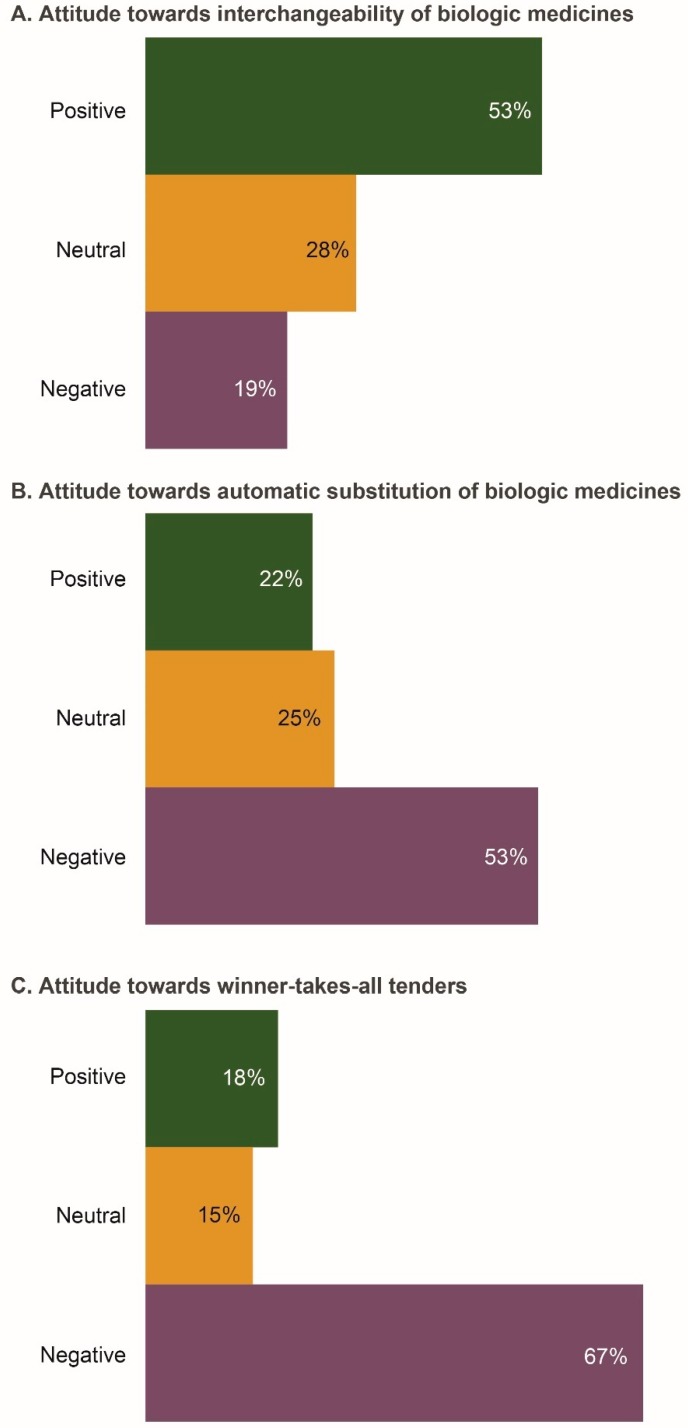
Attitudes of the Russian physicians surveyed (*n* = 206) towards key policy issues associated with prescribing biosimilars. (**A**) Attitude towards interchangeability of biologic medicines; (**B**) Attitude towards automatic substitution of biologic medicines; (**C**) Attitude towards winner-takes-all tenders. Data were extracted from questions 7a, 7b, and 7d of the questionnaire (see Survey Questionnaire 1).

The majority of respondents (53%) were positive about interchangeability (Figure 2A). The main reasons for this positive attitude were “increasing access to biologics and a greater choice of therapeutic options”. Approximately half of respondents (53%) would be negative if a pharmacist had the ability to substitute a biosimilar in place of a biologic drug without the physician’s approval (Figure 2B). Respondents believed that “the right of the physician to choose the most appropriate medicine for their patient should be preserved”. The primary reason for a negative attitude towards automatic substitution was “the possibility of the biosimilar having lower efficacy and safety compared with the reference product”. Physicians were also concerned that “pharmacovigilance data may be confounded if automatic substitution occurs”. Two-thirds of respondents (67%) felt negative about winner-takes-all tenders; one reason cited for this was the need for physicians to have a choice in selecting the most appropriate medicine for any given patient (Figure 2C). 

The majority of respondents (64%) supported prescribing biologics (including biosimilars) by brand (distinguishable) names, to ensure traceability of adverse events. Some physicians highlighted that “brand names are important to ensure that the patient receives the same drug as previously prescribed and are not switched to another biologic at the dispensing level”. Additionally, there was concern that “biosimilars might have differing efficacy and safety profiles; thus, biologics should be prescribed by brand names”. The number of respondents who were neutral or positive about INN prescription was relatively low (20% and 16%, respectively). Reasons for a positive attitude to INN prescription included “consistency with Russian regulations” and “supporting the development and manufacturing of medicines in Russia”. 

Further opinions on issues of brand-name biologics and biosimilars are shown in Table 1. Only 57% of respondents agreed/strongly agreed that they were generally comfortable with using biologics; this was even lower (38%; *p* = 0.013) among oncologists. Although 58% of respondents cited that it was difficult to obtain information on clinical efficacy and safety of a biosimilar, 68% trusted that the Russian Ministry of Health approved only efficacious and safe medications. Despite this, 51% believed the risk of side effects was greater for a biosimilar versus the reference product. The majority of respondents agreed/strongly agreed that the publication of clinical study reports should be mandatory for biosimilars (94%), and that biosimilars should be subject to rigorous post-marketing surveillance (98%). 

### 3.4. Guiding Factors for the Use of Biosimilar Products 

The most important factors to guide decisions about biosimilar use in clinical practice among all respondents were: comparative clinical trials between the biosimilar and its reference product (68%), inclusion of a biosimilar in clinical guidelines and standards of treatments (55%), and comparative immunogenicity data (42%) (Table 2). The most important factors for deciding to use biosimilars by specialty were: for hematologists, comparative clinical trials (86%; *p* = 0.002); for rheumatologists, comparative immunogenicity data (59%; *p* = 0.027); and for oncologists, cost of treatment (46%; *p* = 0.048; marginally significant). 

### 3.5. Issues Related to Biosimilars in Professional Environments 

Access to clinical trial results (54%) and approaches to interchangeability and automatic substitution (53%) were the top two priorities related to biosimilar use in a professional environment (Table 3). The least important issues by specialty were: for rheumatologists, tender policy (8%; *p* = 0.013); for hematologists, switching (6%; *p* = 0.003); and for oncologists, integration into clinical practice (6%; *p* = 0.004).

### 3.6. Need for Biosimilars Education and Preferred Educational Format 

Almost all respondents (94%) expressed a need for further education related to biosimilars (Figure S3). The proportion of gastroenterologists who assessed themselves as well informed about biosimilars was significantly lower vs. other specialties (13%; *p* = 0.014). The most common sources of information about biosimilars were: conferences and other live meetings (77%); published literature (in native language; 69%); medical representatives or events organized by pharmaceutical company (68%); internet (61%); and colleagues (49%). A similar distribution of responses was observed when respondents were asked about their preferred education format for learning more about biosimilars. 

### 3.7. Main Characteristics of Physician Groups Based on Their Knowledge of Biosimilars 

Physicians were divided into three groups, depending on how they answered two questions that assessed their understanding of the differences between biosimilars and generic drugs/intended copies. Only 20% provided correct answers for both questions (group 1), 33% gave the correct answer once or selected the “do not know” option (group 2), and 47% provided incorrect answers twice (group 3). The distribution of physicians in these groups was similar across all specialties, with a few exceptions. Compared with groups 1 and 3, respectively, group 2 had a higher proportion of rheumatologists (37% vs. 17% (*p* = 0.012) and 20% (*p* = 0.013)), but a numerically lower proportion of hematologists (15% vs. 31% (*p* = 0.055) and 28% (*p* = 0.040)).

Numerically fewer respondents in group 3 worked in Moscow: 29% vs. 43% in group 1 (*p* = 0.117) and 42% in group 2 (*p* = 0.088). Furthermore, 8% in group 3 stated they have a “great need to learn more about biosimilars” compared with 29% in group 1 (*p* = 0.007) and 28% in group 2 (*p* = 0.001). A higher proportion of respondents in group 3 believed that a biosimilar might be a medicine that is not necessarily developed in line with a strictly comparative development program: 14% vs. 0% (*p* < 0.001) and 4% (*p* = 0.023) in groups 1 and 2, respectively. More physicians from group 3 looked for information in published literature rather than publications about biosimilars available on the internet. Numerically more respondents in group 1 had negative attitudes towards automatic substitution compared with groups 2 and 3: 67% vs. 46% (*p* = 0.032) and 51% (*p* = 0.069), respectively. However, physicians in group 1 were generally positive about the introduction of biosimilars in Russia.

All groups had a lack of understanding of the patient profile most appropriate for treatment with biosimilars, were equally concerned about preferences for local manufacturers in tenders, and were more likely to trust innovative drugs rather than biosimilars, as they believed that the former are more efficacious and safe. 

## 4. Discussion

A number of studies have been conducted in different countries, and among members of different medical societies, to assess physician knowledge and attitudes towards biosimilars [29,30,31,36]. However, this is the first study to evaluate the perception of physicians in Russia on key areas surrounding biosimilars. Based on these analyses, a significant proportion of Russian physicians across specialties (rheumatology, gastroenterology, hematology, and oncology) lack confidence in prescribing biologic therapies to their patients. Although there was a preference for reference products compared with biosimilars, most respondents were positive towards the introduction of biosimilars in Russia, as they believed biosimilars could potentially increase access to biologic therapies and provide more treatment options for patients.

The targeted responders included oncologists, hematologists (including hematologist-oncologists), rheumatologists, and gastroenterologists (i.e., specialists who are mostly involved in the management of patients with biologic monoclonal antibody therapies). Compared with other specialists in this survey, the higher number of hematologists prescribing biologic medicines could be a result of the full reimbursement of treatments for hematologic diseases, thereby avoiding issues of accessing medications. This is not the case for gastroenterology, rheumatology or oncology. The lower proportion of gastroenterologists prescribing biologic medicines could be explained by the lack of approved biosimilars for gastroenterology in the Russian market; at the time the survey was conducted, biosimilar infliximab (Celltrion; Incheon, South Korea) had been approved, but was not yet launched, for the treatment of inflammatory bowel disease [37]. 

There is a large unmet need for general education regarding biosimilars among physicians of the surveyed specialties in Russia. The majority of physicians demonstrated a lack of knowledge about the differences between biosimilars and generic medicines, as well as regulatory policies surrounding biosimilars. This is in contrast to a survey of physicians conducted in Canada, which indicated 89% of respondents appreciated the differences between generic drugs and biosimilars [30]. One reason for the low familiarity with biosimilars in Russia is the lack of biosimilar regulation and associated documents/guidance published by Russian healthcare authorities. However, the findings reported here indicate that Russian physicians are interested in learning about biosimilars. 

The majority of respondents believed the publication of clinical trial results should be mandatory for all approved products. Furthermore, almost all physicians believed that biosimilars should be subject to rigorous post-marketing surveillance, including establishing patient registries, in order to provide further reassurance on the safety and tolerability of biosimilars. These results are concordant with a US survey, wherein respondents placed studies that directly compared clinical efficacy and safety between reference products and biosimilars as most important in helping to inform decisions about biosimilar use [31]. 

Overall, there was a poor understanding of the issues related to interchangeability and automatic substitution of biologic medicines in Russia. The majority of survey respondents believed that biologics, including biosimilars, should be purchased and prescribed by brand (distinguishable) names, and were negative about automatic substitution and winner-takes-all tenders. Additionally, more physicians were negative about substitution than about interchangeability (53% vs. 19%, respectively). This discrepancy may indicate a lack of understanding of biosimilar policies among Russian physicians, as well as the fear of a possible drug substitution at the pharmacy level without the physician being informed. Indeed, to date, there are no consolidated position statements from Russian medical communities on the issue of interchangeability. “Greater familiarity with established brand-name drugs” and “uncertainty over the long-term safety of biosimilars” were often given as reasons for not offering biosimilars to patients. 

In the current survey, oncologists were less concerned about biosimilar introduction in clinical practice compared with other specialties. One possibility for this is the shorter courses of treatment for oncology patients; therefore, oncologists may be less concerned about issues related to immunogenicity, and any associated secondary loss of response to treatment. Additionally, it is possible that oncologists will more readily accept a biosimilar because of poor access to biologic oncology medicines in Russia [38,39,40,41]. Hematologists placed less importance on issues surrounding switching between reference biologics and biosimilars. Due to a singular source of tender, rituximab was the first monoclonal antibody drug substituted with the locally developed rituximab biosimilar in Russian patients. Therefore, the results may reflect that hematologists are satisfied with the local rituximab biosimilar or that they do not have access to post-marketing surveillance data. Since regional authorities in Russia can independently change their policies concerning replacement of drugs, and more patients are likely to be switched from reference products to biosimilars in the future, there is a need for further guidance on interchangeability and substitution in Russia, as well as additional policies for clinical-trial reporting and pharmacovigilance. 

There are a number of limitations associated with this analysis. Firstly, the population was limited to physicians who had internet access and who were identified from the database; nevertheless, since the sample includes physicians from most institutions authorized to specialize in biological therapy, this population can be considered representative of Russian physicians prescribing biologics. Secondly, respondents were not analyzed by length of clinical practice. Thirdly, the respondents’ subjective (self-assessment) answers may not reflect their clinical practice. Many of the Russian physicians who participated in the survey will only have had experience with locally developed biosimilars; therefore, another limitation is that the study findings cannot be extrapolated to biosimilars produced by international manufacturers and approved in well-regulated markets. 

## 5. Conclusions

This is the first attempt to survey the knowledge and attitudes of Russian physicians towards biosimilars and the policies related their regulatory approval and use in Russia. While respondents to the survey broadly recognized the potential benefits biosimilars could offer in terms of increasing patient access to biologic treatments, understanding was lacking amongst some physicians concerning the relationship of a biosimilar to its reference biologic and the policies that were in place in Russia supporting their authorization and use. Amongst the most important identified factors related to biosimilars to better support treatment decision making, was the need for the availability of evidence from comparative clinical trials of biosimilars versus their reference product (including PK data and immunogenicity findings), as well as the inclusion of biosimilars in the relevant treatment guidelines. The study results highlight the unmet needs for biosimilar education in this region. Current physician attitudes towards, and perceptions of, biosimilars can inform future educational initiatives and highlight important issues for payers, policymakers and other stakeholders, to shape balanced and evidence-based policies for biologic medicines in Russia. 

## Figures and Tables

**Table 1:** Attitudes and perceptions of physicians who responded to the survey about brand-name biologics and biosimilars (*n* = 206).

Statement	Agree/Strongly Agree
I am generally comfortable prescribing biologic drugs to my patients	57%
If a drug has been approved by the Russian Ministry of Health, I would offer it to my patients because I am confident it is safe and efficacious	68%
Biosimilars are essentially the same as generic drugs	55%
Usually it is difficult to obtain information on clinical efficacy and safety for a biosimilar	58%
Biosimilars clinical trial data should be included in labeling to guide physician and patient decisions	93%
Publication (transparency) of clinical trial reports for biosimilars should be mandatory	94%
The risk for side effects is greater with a biosimilar than for the reference product	51%
Biosimilars should be subject to rigorous post-marketing surveillance, including establishing efficient patient registries	98%
Biosimilars will have a significant impact on clinical practice in Russia for another 3–5 years	83%
I would feel comfortable prescribing biosimilars if I am confident in their quality, efficacy, safety, and similar immunogenicity against the reference product	91%

Data were extracted from question 8 of the questionnaire (see Survey Questionnaire 1).

**Table 2:** The importance of types of information for making decisions to use biosimilar products (*n* = 206).

Statement	Important/Extremely Important	Selected as Being in the Three Most Important Statements
Studies that provide clinical immunogenicity data for the biosimilar and reference product	97%	42%
Studies that directly compare clinical efficacy and safety between reference products and biosimilars	96%	68%
Studies that show pharmacokinetic similarities between reference products and biosimilars	96%	30%
Inclusion in international and Russian clinical practice guidelines and standards of treatment	95%	55%
Studies that show chemical/physical similarities between reference products and biosimilars	89%	24%
Studies that compare activity with in vitro functional assays between reference products and biosimilars	87%	21%
Acquisition cost differences	78%	31%
Colleague and expert opinion	78%	8%
Payer decisions and requirements	69%	21%

Data were extracted from question 10 of the questionnaire (see Survey Questionnaire 1).

**Table 3:** The importance of issues related to biosimilars in professional environments (*n* = 206).

Statement	Important/Extremely Important	Selected as Being in the Three Most Important Statements
Tracking safety events with biosimilars	99%	49%
Access to information on studies comparing biosimilars with reference biologics	96%	54%
Establish reasonable and scientifically justified approach to interchangeability and automatic substitution	93%	53%
Physician authority to decide on the most suitable biologic for each patient	89%	47%
Knowledge about biosimilars among interdisciplinary colleagues	86%	24%
Preparing (educating about biosimilars, which includes patients) to integrate biosimilars into clinical practice	84%	18%
Switching between reference biologics and biosimilars	74%	19%
Naming conventions for biosimilars (unique vs. same non-proprietary names)	74%	16%
Tender policy with preference for Russian manufacturers	54%	19%

Data were extracted from question 11 of the questionnaire (see Survey Questionnaire 1).

## Supplementary Materials

The following are available online at http://www.mdpi.com/2218-273X/9/2/57/s1. Table S1: Biosimilars of biologics in rheumatology and oncology currently available in Russia. Survey Questionnaire 1: English language version of physician survey on biosimilars in Russia. Survey Questionnaire 2: Russian language translation of physician survey on biosimilars in Russia. Figure S1: Prescribing of biologic medicines and familiarity with biosimilars (*n* = 210). Figure S2: Russian physicians included in the analysis. Table S2: Physician knowledge related to approval pathways and related regulatory issues for biosimilars in Russia. Figure S3: Need for education on biosimilars (*n* = 206).
