# Supplementary material for: Evaluation of Physicians’ Knowledge and Attitudes Towards Biosimilars in Russia and Issues Associated with Their Prescribing
Source: Biomolecules. 2019 Feb 11;9(2):57. doi: 10.3390/biom9020057 (PMC6406747; doi:10.3390/biom9020057)
Supplement: Supplementary file 1 [file biomolecules-09-00057-s001.zip › Russian Phys manuscript_Biomolecules_Supp File 1_Table S1_21dec18_1.0.docx]

**Table S1.** Biosimilars of biologics in rheumatology and oncology currently available in Russia.

| INN | Trade name | Approval date in Russia, reg. number | Indications | Manufacturer |
| --- | --- | --- | --- | --- |
| Infliximab | Infliximab | 15.01.2018  *ЛП-004634* | Rheumatoid arthritis, ankylosing spondylitis, psoriatic arthritis, psoriasis, Crohn’s disease, ulcerative colitis | "BIOCAD" |
|  | Flammegis | 06.07.2015  *ЛП-003075* | Rheumatoid arthritis, ankylosing spondylitis, psoriatic arthritis, psoriasis, Crohn’s disease, ulcerative colitis | Celltrion (same as “Remsima”, but distributed by EGIS Pharmaceuticals PLC) |
| Rituximab | Acellbia | 04.04.2014  *ЛП-002420* | Rheumatoid arthritis,, Granulomatosis with polyangiitis, Microscopic polyangiitis, chronic lymphocytic leukemia, non-Hodgkin lymphoma | "BIOCAD" |
|  | Redditux | 25.04.2016  ЛП-003584 | chronic lymphocytic leukemia, non-Hodgkin lymphoma | Dr Reddi’s |
| Trastuzumab | Gerticad | 31.12.2015  ЛП-003403 | mammary cancer,  stomach cancer | "BIOCAD" |
|  | Trastuzumab | 31.12.2015  ЛП-003403 | mammary cancer,  stomach cancer | "BIOCAD" |
| Bevacizumab | Avegra BIOCAD | 25.11.2015  ЛП-003336 | mammary cancer,  pulmonary cancer,  colorectal cancer, ovarian cancer, cervical cancer, glioblastoma | "BIOCAD" |
| Filgrastim | Leucostim | 15.06.2009  ЛС-002011 | neutropenia | "BIOCAD" |
|  | Mielastra | 23.03.2012  ЛС-002703 | neutropenia | Veropharm |
| Epoetin beta | Erythropoietin | 19.12.2011  ЛС-001854 | anemia | "Binnopharm" |
|  | Erythrostim | 20.01.2012 Р N000525/01 | anemia | “Microgen” |
| Epoetin alpha | Binocrit | 30.01.2012  ЛП-001466 | anemia | Sandoz |
