# Supplementary material for: Evaluation of Physicians’ Knowledge and Attitudes Towards Biosimilars in Russia and Issues Associated with Their Prescribing
Source: Biomolecules. 2019 Feb 11;9(2):57. doi: 10.3390/biom9020057 (PMC6406747; doi:10.3390/biom9020057)
Supplement: Supplementary file 1 [file biomolecules-09-00057-s001.zip › Russian Phys manuscript_Biomolecules_Supp File 2_Survey Q1_15jan19_2.0.docx]

**Survey Questionnaire 1.**

English language version of physician survey on biosimilars in Russia

(The survey was translated to Russian language [see Supplementary File 3: Survey Questionnaire 2] and all primary data were collected in Russian).

| **SCREENER** |
| --- |

Dear doctors, the independent survey company Ipsos is conducting a survey to gauge knowledge and understanding on a few topics relevant to biosimilars among rheumatologists, haematologists, oncologists and gastroenterologists.

Despite that biosimilars can help to generate savings for healthcare systems and increase patient access to biologic therapies, considerable controversy remains regarding biosimilars’ regulatory approval rationale and policies associated with their prescribing.

Therefore, we would be very grateful if you could take around 15 minutes to fill out a survey that may help to improve country regulatory policies and establish recommendations for future educational programs. The questionnaire is anonymous, your answers will be used in aggregate form and after statistical processing only; results of the study can be published in scientific journals.

Thank you very much for your time and cooperation!

**S1. Please choose your specialty** /SINGLE ANSWER/

| 1 | Rheumatology | **CONTINUE** |
| --- | --- | --- |
| 2 | Gastroenterology | **CONTINUE** |
| 3 | Haematology | **CONTINUE** |
| 4 | Oncology | **CONTINUE** |
| 5 | Other | **CLOSE** |

**S2. Which of the following medications, if any, did you prescribe to your patients
in the last 12 months?** /MULTIPLE ANSWER/

|  | **PROGR: SHOW IF S1=1 (RHEUMATOLOGY)** |
| --- | --- |
| **1** | **Adalimumab** |
| **2** | **Infliximab** |
| 3 | Methotrexate |
| **4** | **Rituximab** |
| 5 | Sulfasalazine |
| **6** | **Tocilizumab** |
| **7** | **Etanercept** |
|  | **PROGR: SHOW IF S1=2 (GASTROENTEROLOGY)** |
| **10** | **Adalimumab** |
| 11 | Azathioprine |
| **12** | **Golimumab** |
| **13** | **Infliximab** |
| 14 | Methylprednisolone |
| 15 | Methotrexate |
| 16 | Sulfasalazine |
| **17** | **Certolizumab pegol** |
|  | **PROGR: SHOW IF S1=3 (HEMATOLOGY)** |
| 20 | Vincristine |
| 21 | Doxorubicin |
| **22** | **Rituximab** |
| 23 | Fludarabin |
| 24 | Cyclophosphamide |
|  | **PROGR: SHOW IF S1=4 (ONCOLOGY)** |
| **30** | **Bevacizumab** |
| 31 | Gemcitabine |
| 32 | Doxorubicin |
| 33 | Paclitaxel |
| 34 | Tamoxifen |
| **35** | **Trastuzumab** |

**S3. How would you rate your overall familiarity with Biosimilars corresponding to:
1 (never heard of biosimilars) to 5 (very familiar) scale?** /SINGLE ANSWER/

| **1.** Never heard of biosimilars | **2.** I heard about existing of biosimilars only | **3.** Somewhat familiar | **4.** Familiar | **5.** Very familiar/have experience with biosimilars |
| --- | --- | --- | --- | --- |

**NON-PRESCRIBERS of BIOLOGICS & NOT FAMILIAR WITH BIOSIMILARS: -> GO TO Q13 & Q14**

**BIOLOGICS PRESCRIBERS & NOT FAMILIAR** **WITH BIOSIMILARS: -> GO TO Q13 & Q14**

**NON-PRESCRIBERS of BIOLOGICS & FAMILIAR WITH BIOSIMILARS: -> GO TO Q4 and Q13 & Q14**

**BIOLOGICS PRESCRIBERS & FAMILIAR WITH BIOSIMILARS: -> ASK ALL THE QUESTIONS**

| **MAIN QUESTIONNAIRE** |
| --- |

**Q4. Select the correct statement about biosimilars.** /SINGLE ANSWER/

| 1 | Biosimilars are generic versions of the reference biologic |
| --- | --- |
| 2 | Biosimilars are identical copies of the reference biologic |
| 3 | Biosimilars are highly similar to the reference biologic |
| 4 | I don't know |

**Q5. How would you rate your knowledge related to an approval pathway and regulatory issues associated with prescribing biosimilars in Russia?** /SINGLE ANSWER/

| 1 | Not familiar |
| --- | --- |
| 2 | Somewhat familiar |
| 3 | Familiar |
| 4 | Very familiar |

**Q6. Please select the correct statement about biosimilars:** /SINGLE ANSWER/

| 1 | Biosimilar is a medicine that is not necessarily developed in line with a strictly comparative development program against a reference product |
| --- | --- |
| 2 | Biosimilar is copy of a therapeutic protein that may have clinically significant differences in formulation, doses/dosing regimen, efficacy, safety, and immunogenicity |
| 3 | Biosimilar may be a biologic, altered to improve disposition, safety, efficacy, dosing frequency, immunogenicity, or manufacturing attributes against a reference product |
| 4 | Biosimilar is a biological medicinal product that contains a highly similar version of the active substance of its reference biologic medicine (a biopharmaceutical that has already been authorized), and for which there are no clinically meaningful differences in safety, purity, or potency of the product |
| 5 | I don’t know |

**Q7. What are your views on the following topics regarding to biosimilars?**

**7а**. **How do you feel about interchangeability?** /SINGLE ANSWER/

Interchangeability – the medical practice of changing one medicine for another, and having done so, achieving the same clinical effect as the original treatment in a given clinical setting and in any patient.

| 1 | Positive |
| --- | --- |
| 2 | Negative |
| 3 | Neutral |

**Further comments or rationale regarding interchangeability**

|  |
| --- |

**7b. How do you feel about automatic substitution?** /SINGLE ANSWER/

Automatic substitution – when a pharmacist substitutes a different biologic medicine for the prescribed biologic product (branded originator or another biosimilar), with no obligation to inform or gain consent from the treating physician, or the patient

| 1 | Positive |
| --- | --- |
| 2 | Negative |
| 3 | Neutral |

**Further comments or rationale regarding automatic substitution**

|  |
| --- |

**7c. How do you feel about opportunity to purchase and prescribe biologics (including biosimilars) by trade (distinguishable) names?** /SINGLE ANSWER/

| 1 | Positive |
| --- | --- |
| 2 | Negative |
| 3 | Neutral |

**Further comments or rationale regarding trade (distinguishable) names**

|  |
| --- |

**7d. How do you feel about “winner-takes-all” tenders (where only one product – originator or biosimilar – is retained for prescribing)?** /SINGLE ANSWER/

| 1 | Positive |
| --- | --- |
| 2 | Negative |
| 3 | Neutral |

**Further comments or rationale regarding winner-takes-all tenders**

|  |
| --- |

**7e. How do you feel about introduction of biosimilars in /SHOW “RHEUMATOLOGY” IF S1=1, SHOW “GASTROENTEROLOGY” IF S1=2, SHOW “ONCOLOGY” IF S1=3-4/ Russia?** /SINGLE ANSWER/

| 1 | Positive |
| --- | --- |
| 2 | Negative |
| 3 | Neutral |

**Why? Please elaborate**

|  |
| --- |

**Q8. On the scale from 1 to 5, where 1 is “Strongly disagree” and 5 is “Strongly agree”, please indicate your level of agreement with each of the following statements.** /SINGLE ANSWER IN EACH ROW/

|  |  | **Strongly disagree** | **Disagree** | **Neutral** | **Agree** | **Strongly agree** |
| --- | --- | --- | --- | --- | --- | --- |
| 1 | I am generally comfortable prescribing biologic drugs to my patients | 1 | 2 | 3 | 4 | 5 |
| 2 | If a drug has been approved by the Russian МoH, I would offer it to my patients because I am confident it is safe and efficacious | 1 | 2 | 3 | 4 | 5 |
| 3 | Biosimilars are essentially the same as generic drugs | 1 | 2 | 3 | 4 | 5 |
| 4 | Usually it is difficult to obtain information on clinical efficacy and safety for a biosimilar | 1 | 2 | 3 | 4 | 5 |
| 5 | Biosimilars clinical trial data should be included in labeling to guide doctors’ and patients’ decisions | 1 | 2 | 3 | 4 | 5 |
| 6 | Publication (transparency) of clinical trials reports for biosimilars should be mandatory | 1 | 2 | 3 | 4 | 5 |
| 7 | The risk for side effects is greater with a biosimilar than with the reference product | 1 | 2 | 3 | 4 | 5 |
| 8 | Biosimilars should be a subject to rigorous post-marketing surveillance, including establishing efficient patient registries | 1 | 2 | 3 | 4 | 5 |
| 9 | Biosimilars will have a significant impact on clinical practice and how patients are treated in Russia for another 3–5 years | 1 | 2 | 3 | 4 | 5 |
| 10 | All things considered, I would feel comfortable prescribing biosimilars to patients if I am confident in their quality, efficacy, safety and similar immunogenicity against the reference product | 1 | 2 | 3 | 4 | 5 |

MoH, Ministry of Health

**Q9. In which patients would you most likely prescribe biosimilar, if it demonstrates that it is comparable to the brand-name drug?**  /MULTIPLE ANSWER/

| 1 | New patients only |
| --- | --- |
| 2 | Switch patients |
| 3 | Adult patients |
| 4 | Other /SPECIFY/ |

**Q10. On the scale from 1 to 5, where 1 is “Not important at all” and 5 is “Extremely important”, please rank the following types of information by importance in helping you decide to use biosimilar products in your patient population.** /SINGLE ANSWER IN EACH ROW/

|  |  | **Not at all important** | **Not important** | **Neutral** | **Important** | **Extremely important** |
| --- | --- | --- | --- | --- | --- | --- |
| 1 | Studies that directly compare clinical efficacy and safety between reference products and biosimilars | 1 | 2 | 3 | 4 | 5 |
| 2 | Studies that show pharmacokinetic similarities between reference products and biosimilars | 1 | 2 | 3 | 4 | 5 |
| 3 | Studies that show chemical/physical similarities between reference products and biosimilars | 1 | 2 | 3 | 4 | 5 |
| 4 | Inclusion in international and Russian clinical practice guidelines and standards of treatment | 1 | 2 | 3 | 4 | 5 |
| 5 | Studies that provide clinical immunogenicity data for the biosimilar and reference product | 1 | 2 | 3 | 4 | 5 |
| 6 | Studies that compare activity with *in vitro* functional assays between reference products and biosimilars | 1 | 2 | 3 | 4 | 5 |
| 7 | Acquisition cost differences | 1 | 2 | 3 | 4 | 5 |
| 8 | Payer decisions and requirements | 1 | 2 | 3 | 4 | 5 |
| 9 | Colleague and expert opinion | 1 | 2 | 3 | 4 | 5 |

**Ask Q10-1, if more than 3 statements have rates 3-5 in q10. show only these statements**

**Q10-1. Please choose three most important statements.** /THREE ANSWERS ARE MAX/

**Q11. On the scale from 1 to 5, where 1 is “Not important at all” and 5 is “Extremely important”, please rank the importance of each of the following issues related to the use of biosimilars to your professional environment.** /SINGLE ANSWER IN EACH ROW/

|  |  | **Not important at all** | **Not important** | **Neutral** | **Important** | **Extremely important** |
| --- | --- | --- | --- | --- | --- | --- |
| 1 | Access to information on studies comparing biosimilars with reference biologics | 1 | 2 | 3 | 4 | 5 |
| 2 | Knowledge about biosimilars among interdisciplinary colleagues | 1 | 2 | 3 | 4 | 5 |
| 3 | Switching between reference biologics and biosimilars | 1 | 2 | 3 | 4 | 5 |
| 4 | Tracking safety events with biosimilars | 1 | 2 | 3 | 4 | 5 |
| 5 | Preparing to integrate biosimilars into clinical practice including educating patients about biosimilars | 1 | 2 | 3 | 4 | 5 |
| 6 | Physician authority to decide on the most suitable biologic for each patient | 1 | 2 | 3 | 4 | 5 |
| 7 | Establish reasonable and scientifically justified approach to interchangeability and automatic substitution | 1 | 2 | 3 | 4 | 5 |
| 8 | Naming conventions for biosimilars (unique vs. same non-proprietary names) | 1 | 2 | 3 | 4 | 5 |
| 9 | Tender policy with the preferences to local manufacturers | 1 | 2 | 3 | 4 | 5 |

**Ask Q11-1, if more than 3 statements have rates 3-5 in q10. show only these statements**

**11-1. Please choose three most important issues.** /THREE ANSWERS ARE MAX/

**Q12. Where have you received information about biosimilars to date?** /MULTIPLE ANSWER/

| 1 | Conferences/live meetings |
| --- | --- |
| 2 | Published literature |
| 3 | Internet (online education and/or self-study including publications) |
| 4 | Colleagues |
| 5 | Medical representative or event organized by pharmaceutical company |
| 6 | Other /SPECIFY/ |
| 7 | No education on biosimilars to date |

**PROGR: ASK ALL**

**Q13. Which of the following best describes your need for education related to biosimilars?**

/SINGLE ANSWER/

| 1 | I have a great need to learn more about this topic |
| --- | --- |
| 2 | I have a basic understanding of biosimilars, but would like to learn more |
| 3 | I am well informed about biosimilars, but want to learn more as new developments occur |
| 4 | I am satisfied with my current knowledge of this area, no further education needed |

**PROGR: ASK Q14, IF CODE 4 IS NOT MARKED IN Q13**

**Q14. What would be your preferred education format to learn more about biosimilars?** /MULTIPLE ANSWER/

| 1 | National meetings/symposia |
| --- | --- |
| 2 | Regional/local live meetings |
| 3 | Online educational websites |
| 4 | Medical societies’ communications via internet |
| 5 | Monographs or product-specific dossiers |
| 6 | Directly from pharmaceutical companies (medical representative or mailing) |
| 7 | Other /SPECIFY/ |

**Q15. Please provide any additional comments or rationale related to this topic**

|  |
| --- |

|  |
| --- |

**Thank you!**
