# Supplementary material for: Evaluation of Physicians’ Knowledge and Attitudes Towards Biosimilars in Russia and Issues Associated with Their Prescribing
Source: Biomolecules. 2019 Feb 11;9(2):57. doi: 10.3390/biom9020057 (PMC6406747; doi:10.3390/biom9020057)

**Survey Questionnaire 2.**

Russian language translation of physician survey on biosimilars in Russia.


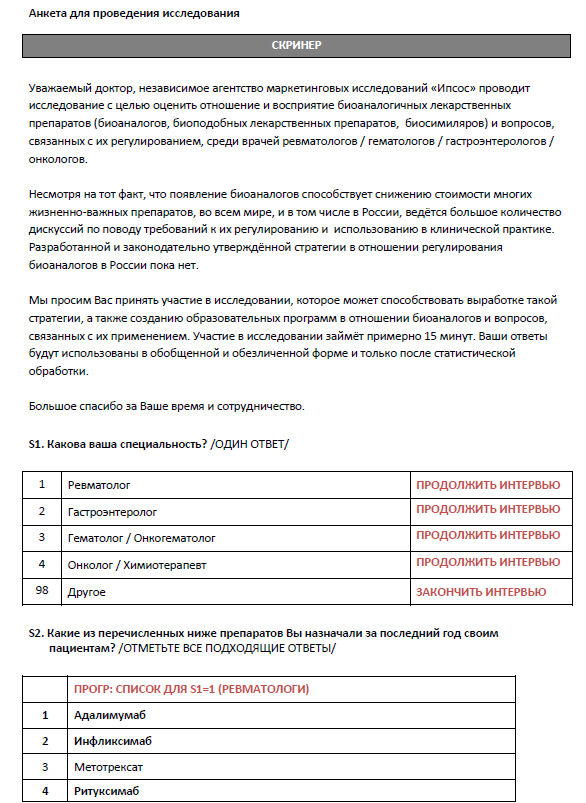


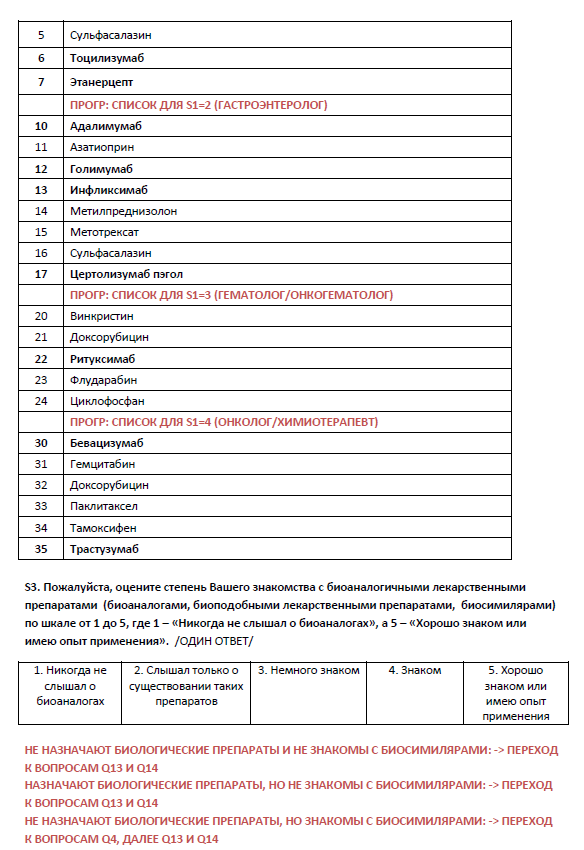


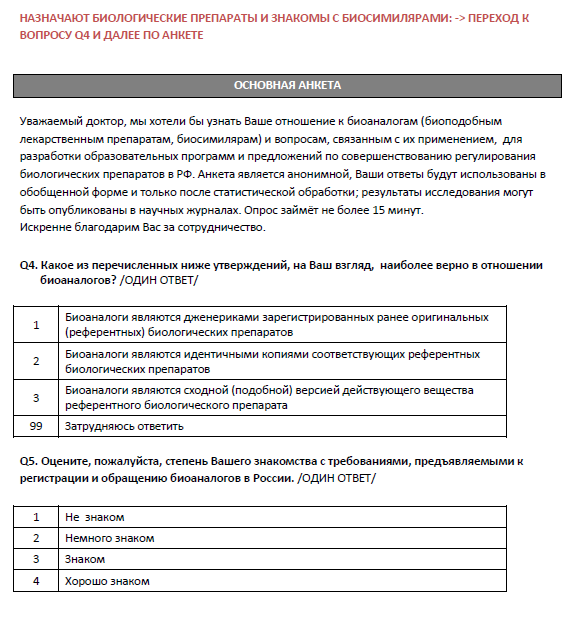


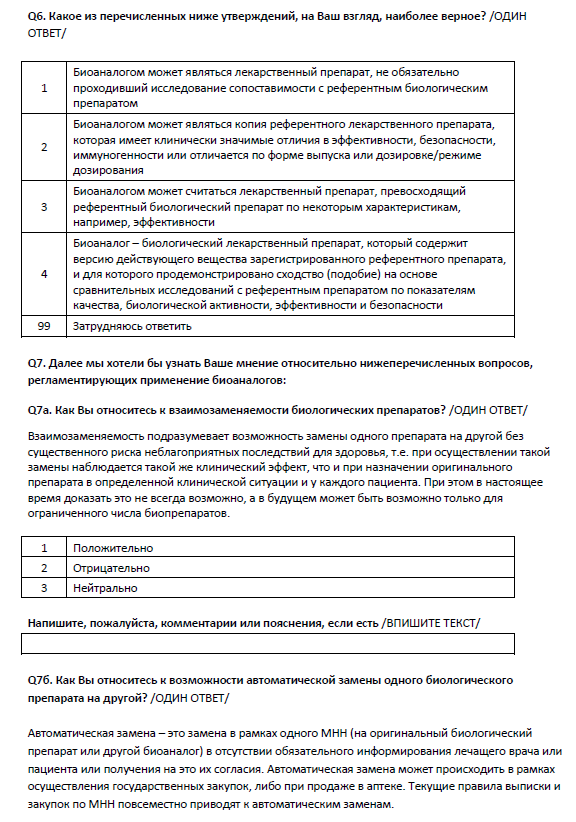


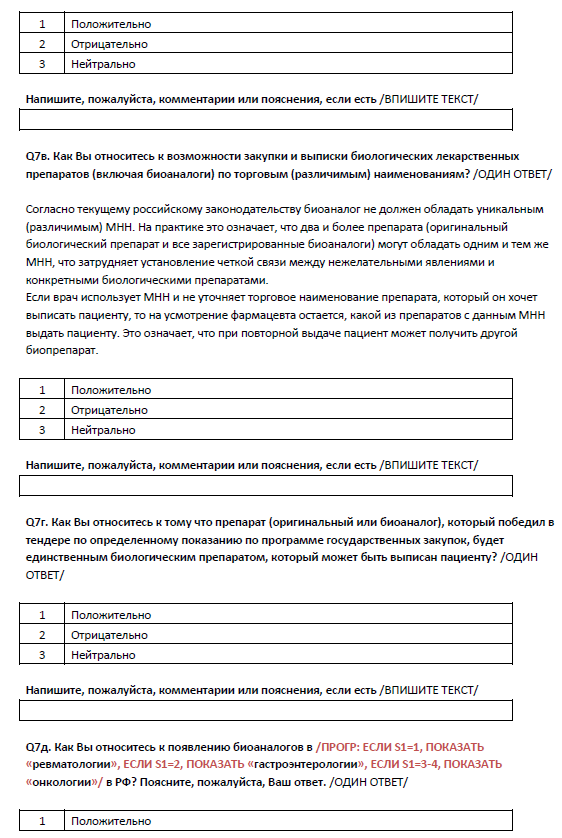


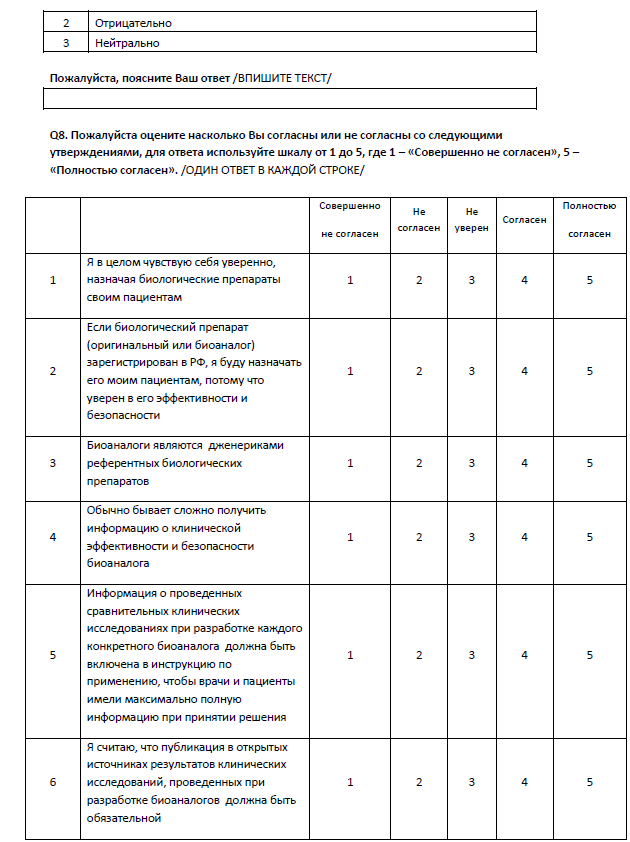


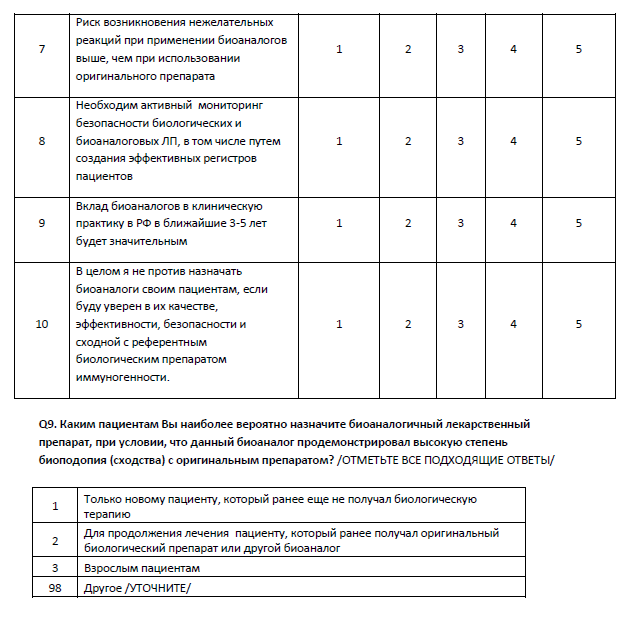


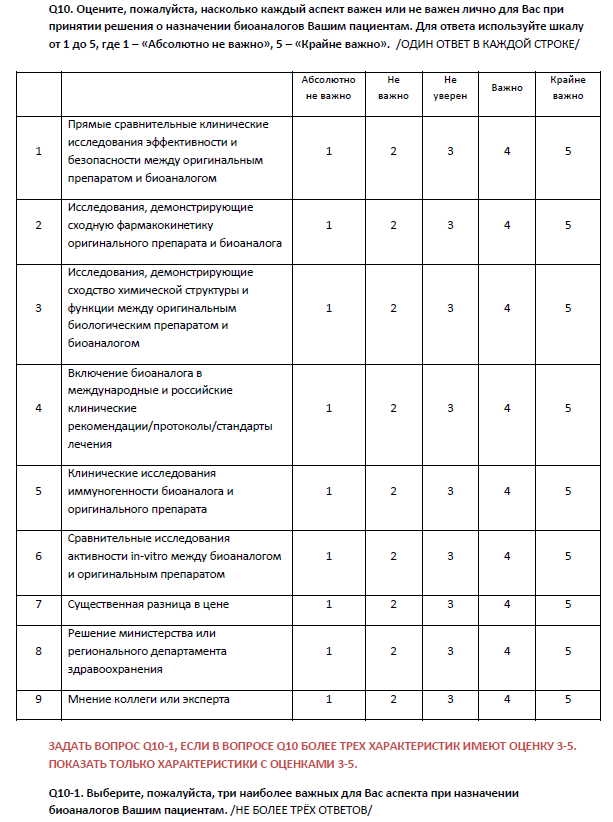


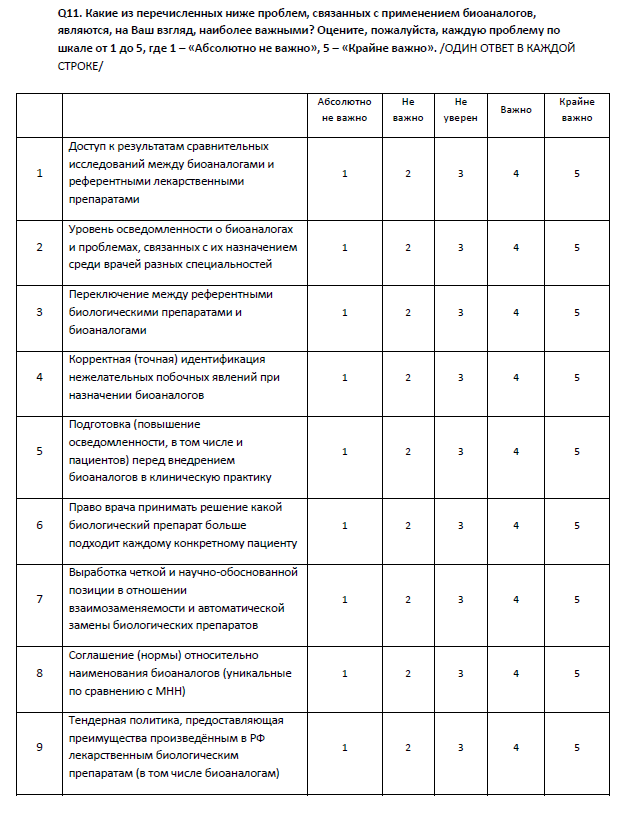


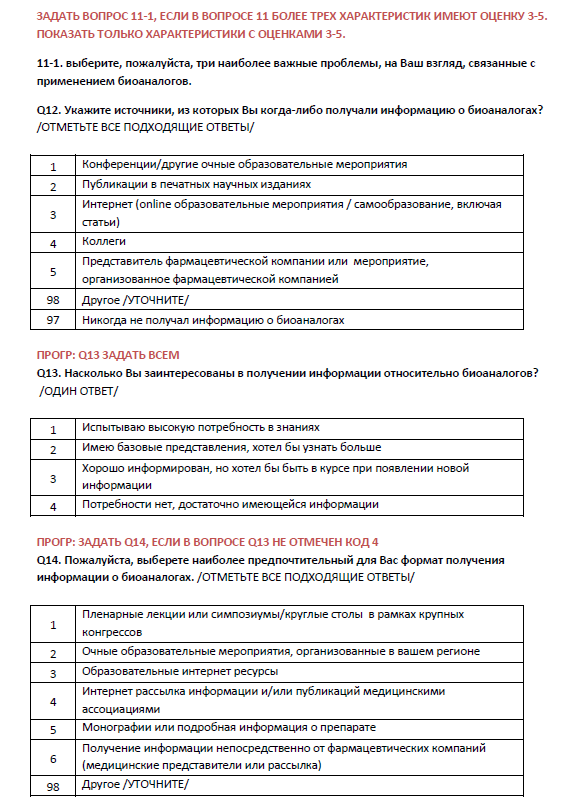


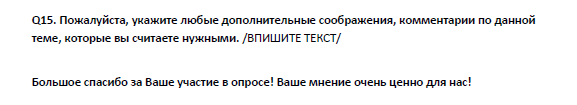

Supplement: Supplementary file 1 [file biomolecules-09-00057-s001.zip › Russian Phys manuscript_Biomolecules_Supp File 3_Survey Q2_21dec18_1.0.docx]
