# Supplementary material for: Evaluation of Physicians’ Knowledge and Attitudes Towards Biosimilars in Russia and Issues Associated with Their Prescribing
Source: Biomolecules. 2019 Feb 11;9(2):57. doi: 10.3390/biom9020057 (PMC6406747; doi:10.3390/biom9020057)
Supplement: Supplementary file 1 [file biomolecules-09-00057-s001.zip › Russian Phys manuscript_Biomolecules_Supp File 4_Figure S1_15jan19_1.0.pdf]

Figure S1. Prescribing of biological medicines and familiarity with biosimilars (N=210)

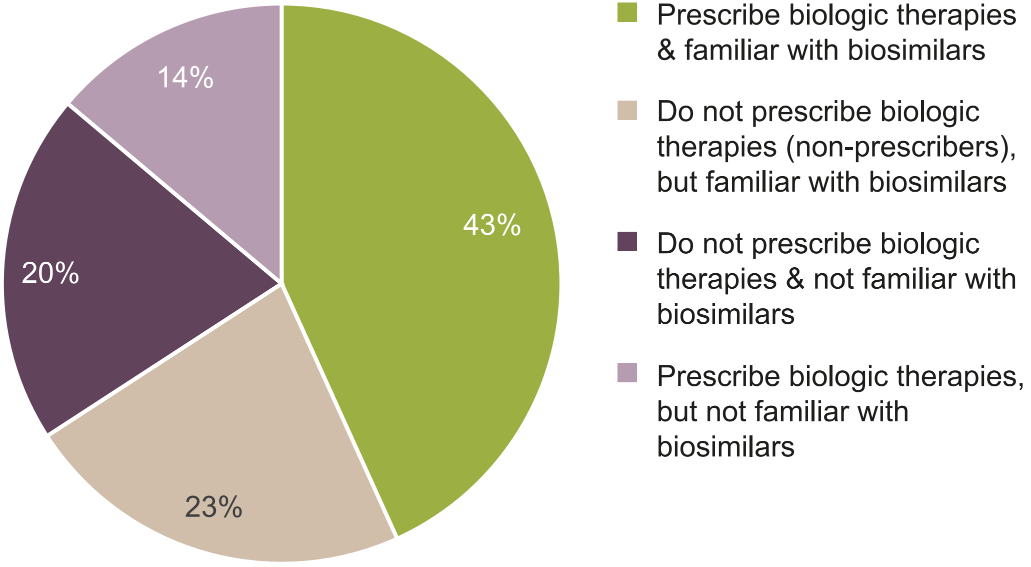

Data were extracted from questions S2 and S3 of the questionnaire (see Supplementary File 2: Survey Questionnaire 1).
