# Supplementary material for: Evaluation of Physicians’ Knowledge and Attitudes Towards Biosimilars in Russia and Issues Associated with Their Prescribing
Source: Biomolecules. 2019 Feb 11;9(2):57. doi: 10.3390/biom9020057 (PMC6406747; doi:10.3390/biom9020057)
Supplement: Supplementary file 1 [file biomolecules-09-00057-s001.zip › Russian Phys manuscript_Biomolecules_Supp File 6_Table S2_15jan19_2.0.docx]

**Table S2.** Physician knowledge related to approval pathways and related regulatory issues for biosimilars in Russia

|  | Total | By specialty (N = 206) | | | | By location (N = 206) | | |
| --- | --- | --- | --- | --- | --- | --- | --- | --- |
|  |  | Rheumatology | Gastroenterology | Hematology | Oncology | Moscow | St Petersburg | Regions |
| n | 206 | 51 | 53 | 50 | 52 | 74 | 33 | 99 |
| Not familiar | 20% | 24% | 32% | 12% | 13% | 27% | 15% | 17% |
| Quite familiar | 34% | 37% | 30% | 36% | 33% | 35% | 45% | 29% |
| Familiar | 39% | 37% | 34% | 46% | 38% | 34% | 33% | 44% |
| Very familiar | 7% | 2% | 4% | 6% | 15% | 4% | 6% | 9% |

Data were extracted from question 5 of the questionnaire (see Supplementary File 2: Survey Questionnaire 1)
