# Supplementary material for: Evaluation of Physicians’ Knowledge and Attitudes Towards Biosimilars in Russia and Issues Associated with Their Prescribing
Source: Biomolecules. 2019 Feb 11;9(2):57. doi: 10.3390/biom9020057 (PMC6406747; doi:10.3390/biom9020057)
Supplement: Supplementary file 1 [file biomolecules-09-00057-s001.zip › Russian Phys manuscript_Biomolecules_Supp File 7_Figure S3_15jan19_1.0.pdf]

Figure S3. Need for education on biosimilars (N=206)

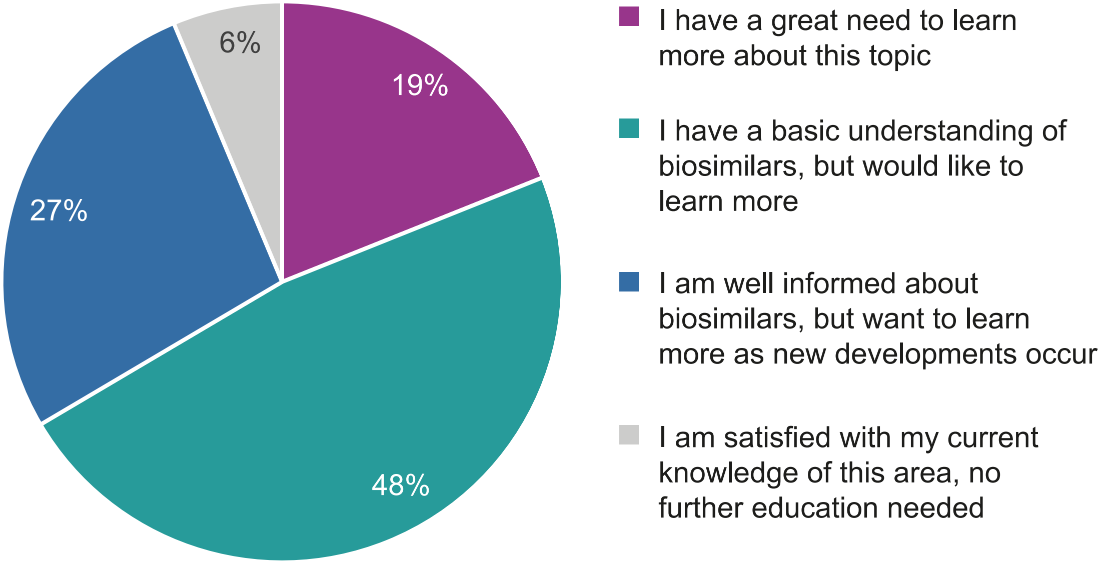

Data were extracted from question 13 of the questionnaire (see Supplementary File 2: Survey Questionnaire 1).
